# Long-latency reflexes account for limb biomechanics through several supraspinal pathways

**DOI:** 10.3389/fnint.2014.00099

**Published:** 2015-01-29

**Authors:** Isaac L. Kurtzer

**Affiliations:** Department of Biomedical Sciences, New York Institute of Technology – College of Osteopathic MedicineOld Westbury, NY, USA

**Keywords:** feedback, posture, internal model, primary motor cortex, cerebellum, reticular formation

## Abstract

Accurate control of body posture is enforced by a multitude of corrective actions operating over a range of time scales. The earliest correction is the short-latency reflex (SLR) which occurs between 20–45 ms following a sudden displacement of the limb and is generated entirely by spinal circuits. In contrast, voluntary reactions are generated by a highly distributed network but at a significantly longer delay after stimulus onset (greater than 100 ms). Between these two epochs is the long-latency reflex (LLR) (around 50–100 ms) which acts more rapidly than voluntary reactions but shares some supraspinal pathways and functional capabilities. In particular, the LLR accounts for the arm’s biomechanical properties rather than only responding to local muscle stretch like the SLR. This paper will review how the LLR accounts for the arm’s biomechanical properties and the supraspinal pathways supporting this ability. Relevant experimental paradigms include clinical studies, non-invasive brain stimulation, neural recordings in monkeys, and human behavioral studies. The sum of this effort indicates that primary motor cortex and reticular formation (RF) contribute to the LLR either by generating or scaling its structured response appropriate for the arm’s biomechanics whereas the cerebellum scales the magnitude of the feedback response. Additional putative pathways are discussed as well as potential research lines.

## Introduction

Barring a neurological disorder or physical impediment, human subjects can accurately position their upper limbs in the presence of unpredictable loads. Consider how one routinely lifts household objects of different weight or can gently secure the wriggling of an anxious newborn. In both the drab and dear situations mechanical perturbations are applied to the arm and require the nervous system to exert compensatory action to ensure task success; the alternative is dropping a cup or child. An important component of our compensation to external loads is the “long-latency reflex” (LLR). First identified over 50 years ago, the LLR is evident as a burst of muscle activity occurring 50–100 ms following an imposed limb displacement. These pre-voluntary responses display an impressive range of capabilities such as integrating sensory information across multiple muscles appropriate for the dynamical interactions between the arm’s linked segments; in this paper I describe this general ability as “knowledge of limb dynamics”. Over two decades of research has demonstrated that LLRs utilize knowledge of limb dynamics and an even more extensive body of research has examined spinal and supraspinal substrates for the LLR. Yet, only recently have these efforts intersected to identify the neural substrates of this capability. Here I review the general features of the LLR, evidence that it utilizes knowledge of limb dynamics, and the relatively small (but growing) research on its neural basis.

## What is the long-latency reflex?

**Figure 1:**
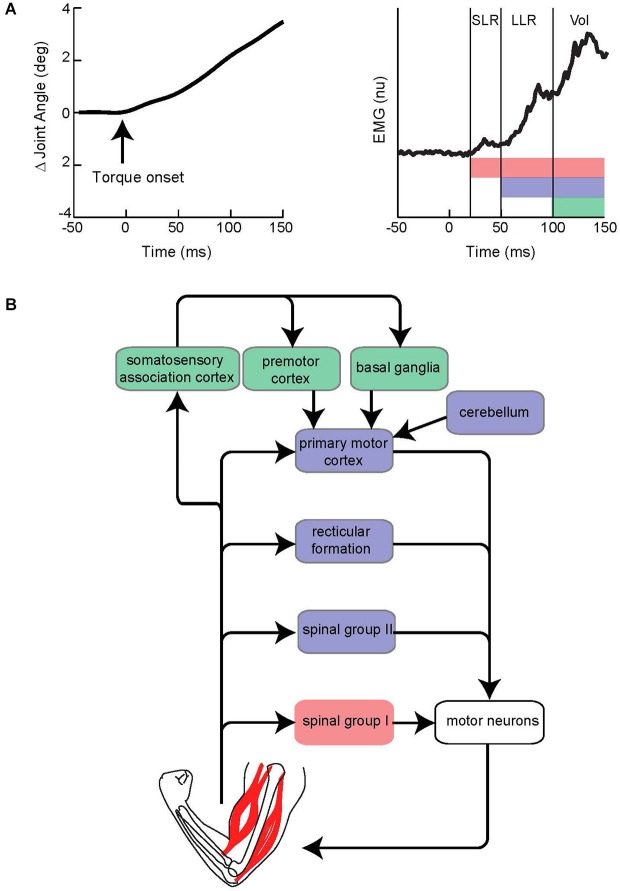
**Evoked muscle activity to limb displacement and proposed neural circuitry. (A)** The left panel depicts an example of joint angle displacement following an applied step torque. The right panel depicts an example of muscle activity evoked by joint displacement. Vertical lines bracket the short-latency reflex (SLR), long-latency reflex (LLR), and Voluntary reaction (Vol) epochs. Pink, purple, and green horizontal bars depict the neural process that contribute to the different epochs. Note the neural contributions continue throughout the perturbation and overlap in time. **(B)** Simplified diagram of neural contributors to the different epochs of evoked activity. Colored boxes correspond to colored bars in panel above. Note that several pathways may be involved for a particular epoch.

This section briefly describes the general characteristics of the LLR. For an extensive treatment see the following reviews (Marsden et al., 1983; Shemmell et al., 2010; Pruszynski and Scott, 2012). The LLR of the upper limb denotes the burst of muscle activity occurring 50–100 ms following a limb displacement. Accordingly, the event is situated between the fastest response by the nervous system termed the short-latency reflex (SLR 20–45 ms) and the more delayed voluntary reaction (100 ms is the earliest onset of a wide distribution) (Figure 1A). The SLR is exclusively generated by spinal networks using group I afferent input as this is only pathway short and fast enough to responsible. The LLR reflects processing of group I afferents through spinal circuits (Hagbarth et al., 1981; Lee and Tatton, 1982; Lewis et al., 2005; Schuurmans et al., 2009; Kurtzer et al., 2010) and supraspinal circuits (including primary motor cortex and reticular formation) (for review Pruszynski and Scott, 2012) along with spinal processing of group II afferents (Hendrie and Lee, 1978; Lourenço et al., 2006; Meskers et al., 2010). Voluntary reactions involve a more distributed circuitry including premotor cortex and basal ganglia as well as the continued impact of faster circuits (Suminski et al., 2007; Shadmehr and Krakauer, 2008). Accordingly, the LLR should not be considered a singular event reflecting a singular neural process, but rather the net impact of spinal and supraspinal circuits contributing within the 50–100 ms time-scale (Figure 1B). Note that all these responses can be observed throughout the muscles of the arm (and the leg) although they vary in relative size according to protocol and possibly intrinsic differences in their neural control. The LLR shares features with both the SLR and voluntary reaction. However, it is not identical to either nor can it be considered a simple mix of the SLR and voluntary reaction due to their temporal overlap. One important difference between the responses is that the SLR and LLR rely mostly on information from muscle afferents whereas voluntary reactions can be engaged by a broader range of somatosensory inputs. Anesthetizing the skin or joint afferents has little affect on the SLR and LLR (Bawa and McKenzie, 1981; Cody and Plant, 1989) whereas non-noxious cutaneous stimulation can evoke a voluntary reaction but not earlier responses (Rothwell et al., 1980); some exceptions have been observed with the fingers appropriate for their specialized role in handling objects (Loo and McCloskey, 1985). Differences between the SLR and LLR are also evident with protocols that selectively attenuate one response but not the other. The tendon tap commonly used during a physical exam will powerfully recruit the SLR but not the LLR (Jaeger et al., 1982; Lee and Tatton, 1982). Conversely, a slow sustained displacement of the limb segment will evoke a substantially larger LLR than SLR (Lee and Tatton, 1982). This period-specific pattern likely reflects differences in their peripheral afferents and central circuits.

An important functional difference between the three epochs is their automaticity (see Pruszynski et al., 2008 for review). Voluntary reactions can be completed suppressed at the whim of the subject whereas SLRs occur in the absence of any voluntary effort and do not change with the intention to react more or less vigorously. LLRs are neither strictly automatic nor strictly voluntary. Subjects attempting to “yield” to a limb displacement will continue to exhibit a LLR, yet they also exhibit larger LLRs when they attempt to “resist” the limb perturbation. We discuss the likely underpinnings of this modifiability in a subsequent section (“Contributions by the reticular formation”). In addition, and central to this review, the the SLR, LLR, and voluntary reaction differ in their ability to integrate sensory information. Voluntary reactions can involve virtually arbitrary couplings of controllable body parts so we can easily flick a finger in response to a tap of the foot. At the other end, SLRs have the least flexible relation to sensory input; they are only evoked in a particular muscle by joint displacements which stretch that muscle. LLRs have a degree of flexibility between the SLR and voluntary reaction as they are evoked by either local muscle stretch or stretch of remote muscles and this mapping accounts for the arm’s biomechanics, in contrast to the near-arbitrary mapping of voluntary reactions.

In sum, the SLR, LLR, and Voluntary responses have a complex partially overlapping character which transitions from the simplest and most rapid to the most complex and most delayed. As a general heuristic we can view these responses as an evolving approximation to the ideal or optimal response with a trade-off between speed and accuracy (Todorov and Jordan, 2002; Scott, 2004). The LLR is a key link in this sequence as it occurs at ≥2X the rate of voluntary reactions and displays a wide range of abilities including knowledge of limb dynamics.

## Why is knowledge of limb dynamics important?

Our bodies are mechanically complex. Movements of one body part depends on applied loads at different body parts due to their physically linkage. Furthermore, this relation is non-linear, position-dependent due to gravity, and context-dependent (e.g., reaching with or without a hand-held object). Actions performed without anticipating this complexity would be inefficient and potentially destabilizing by inducing a series of unwanted and unexpected consequences. Hence, researchers have been highly motivated to understand whether subjects anticipate this complexity and how so. For self-selected actions,the answer is a resounding “yes”;for an extensive treatment see the following reviews (Kawato and Wolpert, 1998; Sabes, 2000; Tin and Poon, 2005). One demonstration (of many) is that healthy subjects can easily acquire an object that is placed on the opposite side of their body by simultaneously turning their trunk and reaching to the object (Pigeon et al., 2003). The twisting trunk movement creates a rotating platform for their arm movements that acts to perturb the path of the hand as it travels to the target. Without the properly counteracting arm torques the hand path would bow outward from the body as the trunk rotated away from it. Instead, our hand movements follow a straight course in external space as if the trunk had not rotated at all. The nervous system achieves this fast and accurate pattern of movement by predictively generating the appropriate counteracting torque since arm movements are significantly disturbed by much smaller perturbations introduced by passively rotating the body while reaching (Lackner and Dizio, 1994) or applying rotary-like forces by a robot (Shadmehr and Mussa-Ivaldi, 1994). Moreover, subjects quickly adapt to such novel forces patterns and then exhibit an opposing pattern of movement errors when the novel forces are removed. The presence of adaptation “aftereffects” indicate an updating of the neural representation of arm dynamics and are a powerful window into the structure of this knowledge.

An two-decade effort has examined how neural representations of limb dynamics are used during self-initiated/planned actions. A number of control schemes have been proposed such as rule-based coordinative patterns (Almeida et al., 1995; Gottlieb et al., 1997), forward models which predict how motor commands create body motion (Flanagan and Wing, 1997), inverse models that transform intended body motions to motor commands (Shidara et al., 1993), paired inverse and forward models (Wolpert and Kawato, 1998), controllers that are collections of local tuning functions (Thoroughman and Shadmehr, 2000) or that identify the underlying physical laws and contexts (Braun et al., 2009). All of these schemes fall under the broad banner of “knowledge of limb dynamics” insofar as they encapsulate (in various degrees) the mechanical properties of the body and environment.

In recent years, researchers have increasingly asked whether our corrective actions also depends on a knowledge of limb dynamics (Diedrichsen, 2007; Wagner and Smith, 2008) in contrast to earlier theories positing that feedback corrections were either simple local corrections or dominated by passive muscle properties (St-Onge et al., 1997; Gribble et al., 1998). Among the accumulating evidence is that fast manual adjustments to a visual target jump (≈125 ms delay in hand position) account for the arm’s complex mechanics and feedback delays (Gritsenko et al., 2009), without this knowledge the hand’s path would be dramatically more curved and irregular than observed. Accordingly, the field of human sensori-motor control is undergoing a significant shift in understanding the relative capabilities of anticipatory/feedforward control vs. corrective/feedback control. Research on the LLR makes an important contribution to this work-in-progress as it is the fastest response by the nervous system (50 ms delay in muscle activity) which utilizes knowledge of limb dynamics.

## Evidence that the long-latency reflex utilizes knowledge of limb dynamics

Studies over the past 30 years have demonstrated that LLRs utilize knowledge of limb dynamics. Here we review some of that evidence. A clear example is the evoked activity of elbow muscles upon forcibly pronating the wrist (Gielen et al., 1988). This perturbation evokes a SLR in the biceps brachii but not brachialis. Biceps brachii is both an elbow flexor and wrist supinator so it is stretched by wrist pronation whereas brachialis is a pure elbow flexor which is neither stretched nor shortened by wrist pronation. Importantly, the perturbation evokes an excitatory LLR in biceps brachii and an inhibitory LLR in brachialis. A decrease in brachialis activation is functionally meaningful since it helps balance the elbow flexion produced by biceps brachii as it counters the applied pronation. If the activity of brachialis was not appropriately decreased, then the arm would generate excessive elbow flexion to a pronating perturbation. Hence, the LLR incorporates information across different muscles appropriate to their mechanical linkage and exemplifies knowledge of this relation.

Serial connection between different segments enables their mechanical interaction so that torque applied at one joint will create motion at that joint and at neighboring joints. A well-studied example is arm movement restricted to elbow and shoulder motion in a single plane (Hollerbach and Flash, 1982; Graham et al., 2003). Applying flexion torque to the shoulder will induce flexion motion of the shoulder along with extension motion of the elbow. Similarly, extension torque applied to the elbow will induce extension motion of the elbow along with flexion motion of the shoulder. Because of these mechanical interactions between the two joints, there is no unique relation between shoulder-elbow torque and motion of a particular joint. Different elbow-shoulder torques can induce the same pattern of motion in a particular joint and the same stretch pattern in a particular muscle. If the arm’s LLRs accounted for these interactions, then the neural networks would integrate information across different muscles appropriate to counter the underlying torque perturbation. Alternatively, if the LLRs did not account for these interactions, then each muscle would exclusively respond to its own stretch.

In a seminal paper, Soechting and Lacquaniti (1988) tested between these possibilities by displacing the shoulder and elbow in various directions via force pulses applied to the hand. They then compared the evoked activity of several arm muscles to the induced shoulder-elbow motion and the derived shoulder-elbow torque. Overall, the LLRs showed a better match to the joint torque opposite the muscle’s action whereas SLRs better matched the joint motion stretching that muscle. Unfortunately, the stream of force pulses induced complex time-varying patterns of joint motion, torque, and background muscle activity which prevented a simple test whether the reflexes responded to local muscle stretch or incorporated stretch information across multiple muscles appropriate for the arm’s biomechanics.

**Figure 2:**
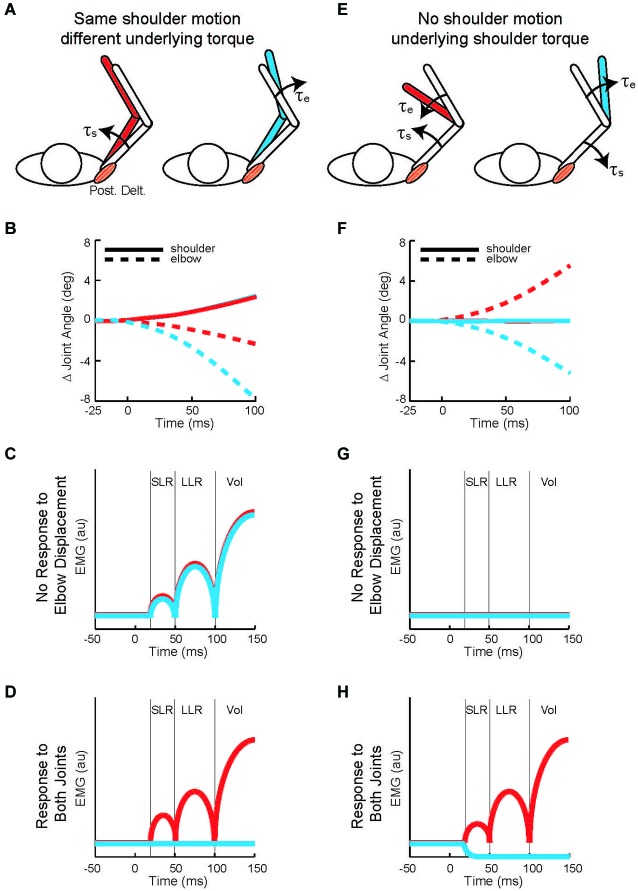
**Testing whether shoulder responses are linked to local muscle stretch or multi-muscle stretch. (A)** Torque perturbations applied to the arm, a shoulder flexor torque (see the red arm) and an elbow extensor torque (see the blue arm). **(B)** Change in joint angle from the starting posture. Solid and dashed lines denote the change in shoulder and elbow angle, respectively. Red and blue indicate motion resulting from shoulder flexor torque and elbow extensor torque, respectively. 0 ms is perturbation onset. Shoulder motion is nearly identical for the two conditions, flexion is positive. **(C)** Predicted shoulder muscle response to the shoulder torque and elbow torque perturbations if the neural processes only utilized local muscle stretch. **(D)** Predicted shoulder muscle responses if the neural processes integrated stretch from shoulder and elbow muscles appropriate to counter the underlying torque. **(E)** Torque perturbations applied to the arm, a shoulder-elbow flexor torque (see the red arm) and a shoulder-elbow extensor torque (see the blue arm). **(F)** Change in joint angle from the starting posture. Same format as **(B)**. The initial joint motion is almost entirely restricted to the elbow. **(G)** Predicted shoulder muscle response to the shoulder torque and elbow torque perturbations if the neural processes only utilized local muscle stretch. **(H)** Predicted shoulder muscle responses if the neural processes integrated stretch from shoulder and elbow muscles appropriate to counter the underlying torque. **(A,B)**, **(E,F)** modified with permission from Kurtzer et al. (2008).

**Figure 3:**
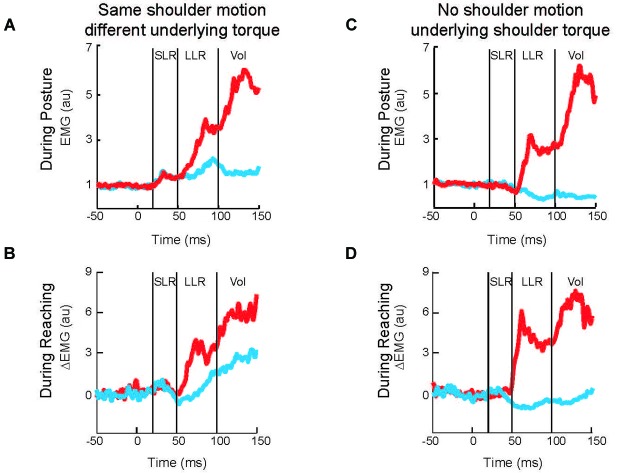
**Shoulder muscle responses to perturbations causing selective joint motion. (A)** Group average of shoulder extensor muscle activity evoked by two perturbations during postural maintenance. Red and blue traces denote activity during shoulder flexor torque and elbow extensor torque perturbations, respectively (Figures 2A,B). **(B)** Group average of shoulder extensor muscle activity evoked by same two perturbations applied during movement initiation; unperturbed pattern of muscle activity has been removed. **(C)** Group average of shoulder extensor muscle activity evoked by two perturbations during postural maintenance. Red and blue traces denote activity during combined flexor and combined extensor torque perturbations which cause elbow flexion and extension, respectively (Figures 2E,F). **(D)** Group average of shoulder extensor muscle activity evoked by same two perturbations applied during movement initiation; unperturbed pattern of muscle activity has been removed. **(A,B)** modified with permission from Kurtzer et al. (2008). **(C,D)** modified with permission from Kurtzer et al. (2009).

Twenty years later Kurtzer et al. (2008) built on this earlier work. Instead of continuous force pulses to the hand, they employed a joint-based robot so that torque could be selectively applied to the shoulder and/or elbow joint (Scott, 1999; Singh and Scott, 2003). The paradigm was focused on posture-related activity of shoulder muscles and contrasted simple patterns of joint motion. In the first set of comparisons, a step torque was applied at just the shoulder or just the elbow (Figure 2A). The relative magnitude of these torque perturbations was chosen to induce the same pattern of shoulder flexion (Figure 2B). That is, because the mechanical interactions between the two joints, the shoulder could be displaced with an elbow torque or a shoulder torque. Inducing the same amount of shoulder flexion (and stretch of the shoulder extensor) allowed a simple model-free comparison between conditions: similar magnitudes of evoked activity indicates processing of only local muscle stretch (Figure 2C) whereas greater evoked activity for the shoulder torque condition indicates compensation of the underlying shoulder torque (Figure 2D). Consistent with the earlier studies, the shoulder displacement evoked an identical SLR in the two conditions but greater LLR following the shoulder torque than elbow torque this pattern was observed for both posture and movement tasks (Figures 3A,B).

In the second set of comparisons, a torque step was applied to both the shoulder and elbow with relative magnitudes (countering the interaction of elbow torque onto shoulder motion) so that only the elbow was displaced (Figure 2E). Shoulder extensor activity that is based on local muscle stretch will not respond to this perturbation since that muscle was neither stretched nor shortened (Figure 2F). Alternatively, shoulder extensor activity that accounted for the mechanical interactions across joints would respond to pure elbow motion to counter the underlying shoulder torque; opposing directions of elbow motion would lead to opposing patterns of excitatory and inhibitory activity (Figure 2G). These perturbations failed to elicit a SLR indicating that it only reflected local muscle stretch, no stretch leading to no response. Reciprocal bursts of activity were present for the shoulder extensor’s LLR which was appropriate for the underlying shoulder torque, an excitatory burst to elbow flexion and inhibitory burst to elbow extension; again, this pattern was observed for both posture and movement tasks (Figures 3C,D). The two sets of comparisons (Same shoulder motion/different underlying torque and No shoulder motion/underlying shoulder torque) provide clear evidence that the LLR, but not the SLR, of shoulder muscles have knowledge of the inertial coupling between the elbow and shoulder.

Subsequent studies tested the generality of this knowledge of limb dynamics. The described pattern was consistently observed across different behavioral contexts including postural maintenance (Kurtzer et al., 2008), movement initiation, and movement deceleration (Kurtzer et al., 2009). It was also expressed throughout the adult age range (20–70 yrs) (Kurtzer et al., 2013) and with displacements so small that they approached the natural variability of behavior (Crevecoeur et al., 2012). Hence, knowledge of elbow-shoulder dynamics is a general capability of the shoulder muscle’s LLRs. See the following papers for examples of wrist muscle LLRs linked to elbow motion (Koshland et al., 1991; Latash, 2000). Also, note that LLRs of leg muscles indicate that they are not slavishly linked to local stretch. A classic example is the differential activation of ankle muscle LLRs to a translating or tilting platform (Nashner, 1976). The two perturbations induce a similar pattern of ankle displacement but responding to local stretch helps stabilize body posture during translation and destabilizes it during platform rotation by moving the body’s center of mass outside the base of support. Recent studies in this vein, supplemented by computational modeling, have further demonstrated that multi-joint integration of body posture utilize a neural representation of the body’s center of mass (Safavynia and Ting, 2013).

LLRs account for the environment’s mechanical stability in addition to the body’s intrinsic musculoskeletal properties. A car’s brake pedal is mechanically stable since greater forces are required to further depress the pedal and its position restores when the foot steps away. In contrast, a screwdriver is mechanically unstable tool since a misaligned force directed parallel to the screw slot can lead to unrecoverable slippage. Considerable research has established that the nervous system addresses such instabilities by changing how the arm responds to displacements (Mussa-Ivaldi et al., 1985; Burdet et al., 2001; Franklin et al., 2007). This can be achieved by realigning the arm’s orientation (and inertial resistance) to the perturbation direction (Trumbower et al., 2009) as well as co-activating muscles to increase their intrinsic stiffness (Rack and Westbury, 1974) and the automatic scaling of reflexes with background muscle activity (Bedingham and Tatton, 1984; Matthews, 1986; Pruszynski et al., 2009). Even further, LLRs can be modified up and down to the environmental stability for a fixed level of background muscle activity.

Adaptation of LLR sensitivity to environmental stability is evident in a variety of situations. Upper limb LLRs, but not SLRs, are downscaled from normal when subjects behave with a servo-controller that enforces a particular movement independent of the subject’s output. Such attenuated responses occur in both single-joint paradigms (Akazawa et al., 1983; Doemges and Rack, 1992) and multi-joint paradigms (Perreault et al., 2008). Conversely, LLRs are upscaled when subjects maintain a steady posture within an unstable environment like a spring with negative stiffness—spring forces that act in the same direction as position deviations and so amplify the positions deviations (Akazawa et al., 1983; Krutky et al., 2010). Upscaled LLRs help provide additional restoring forces to reinforce stable behavior and can be increasingly upscaled for directions of limb motion that have the greatest instability (Krutky et al., 2010). It should be emphasized that adaptation of LLRs to environmental stability is a general capability of the upper limb and is expressed by finger, wrist, elbow, and shoulder muscles. A final example of the arm’s LLR scaling to environmental instability are reaching movements towards a force field which located in a fixed region of space and involves a constant force directed right or left of the hand movement (Kimura et al., 2006), like reaching out of a car window and experiencing the sudden lateral gust of wind. When subjects expect the direction of the upcoming force field then they scale their LLRs appropriately. LLRs elicited as the hand approaches the force field are upscaled in those muscles which compensate the upcoming force direction. The shoulder flexor has an upscaled response to an impending force field expected to extend the shoulder, and the shoulder extensor has an upscaled response to an impending force field expected to flex the shoulder.

A highly influential demonstration of the neural representation of limb dynamics is rapid adaptation of reaching movements to a novel force environment (Lackner and Dizio, 1994; Shadmehr and Mussa-Ivaldi, 1994; Sainburg et al., 1999). If LLRs have similar neural representations then we should expect that LLRs can be rapidly retrained to new force environments. Shadmehr et al. (Ahmadi-Pajouh et al., 2012) tested this possibility by having subjects adapt to a “curl field”—force proportional to the hand’s speed and orthogonal to its heading—and intermittently perturbed the arm with force pulses. Critically, the perturbations occurred just prior to the cued movement onset since alterations in LLRs evoked during movement could reflect true learning or automatic scaling to the altered muscle activity to compensate the curl field. The results demonstrate that adaptation of LLRs was specific to the structure of the curl force field; LLRs were upscaled to a rightwards force pulse during training with a rightwards curl force but not a leftwards curl field. Despite its important contribution, LLRs were tested prior to movement and, therefore, could not disambiguate if they support movement adaptation or are more generic direction-specific responses.

**Figure 4:**
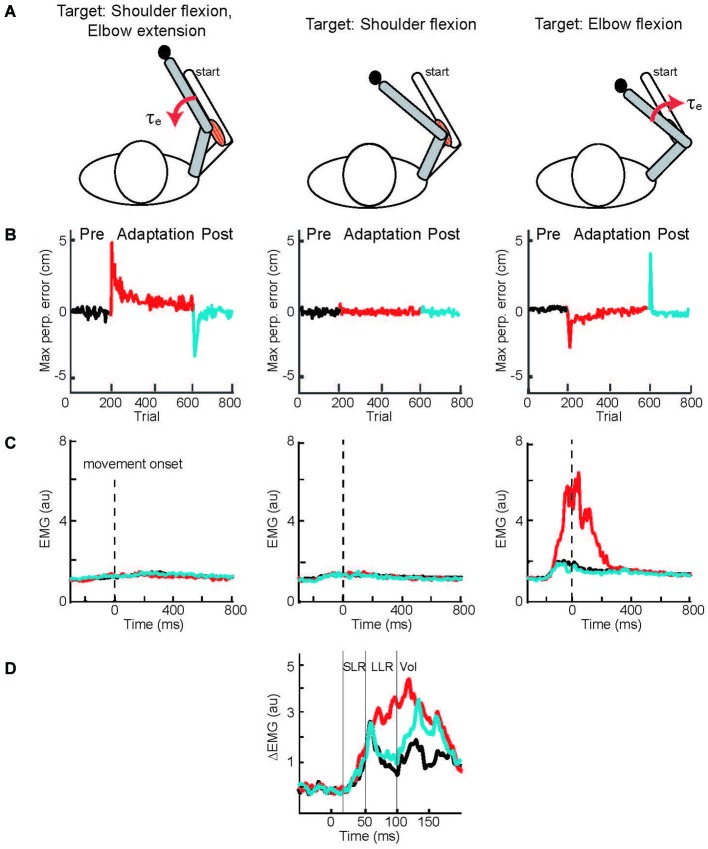
**Applied joint torques and joint motion to test adaptation of LLRs. (A)** Configuration of the arm at the starting position and at the final position when reaching to three targets. A force field applied loads which resisted elbow motion, torque proportional to elbow velocity. The target on the left required shoulder flexion and elbow extension; a resistive load at the elbow applied a flexion torque. The target in the middle only required shoulder flexion; there was no load applied to the elbow as there was no elbow motion. The target on the right required elbow flexion; a resistive load at the elbow applied an extension torque. **(B)** Deviation of the handpaths from a straight line when reaching to the three targets; black, red, and blue denote the movement errors before, during, and after the application of the elbow resistive load. **(C)** Activity of elbow flexor muscle when reaching to the three targets before, during, and after introducing the resistive loads at the elbow. **(D)** Evoked activity of the elbow flexor muscle when reaching to the target requiring only shoulder motion. Data is shown for before, during, and after introducing the resistive load at the elbow. Figure modified with permission from Cluff and Scott (2013).

A solution to this conundrum—how to test LLR adaptation during movement when movement adaptation leads to changes in the compensatory muscle activity and can automatically scale LLRs—was elegantly provided by Cluff and Scott (2013). Rather than a curl force field, subjects adapted their reaching movements to velocity-dependent resistance of their elbow motion (Figure 4A). Successfully moving to targets involving elbow extension and flexion required compensatory activation of elbow extensor and flexor muscles whereas a third target required only shoulder motion so that no compensatory elbow torque was needed. Movement to the targets requiring elbow motion created initial movement errors followed by adaptation (Figure 4B) by changing the anticipatory pattern of elbow muscle activity (Figure 4C). A third target required only shoulder motion so that no compensatory elbow torque was needed and no change in elbow muscle activity was observed (Figures 4B,C). Yet, during movements to this target an elbow extension perturbation evoked upscaled elbow flexor LLRs appropriate to compensate the novel force field (Figure 4D). The link between movement adaptation and LLR adaptation was further strengthened by their similar asymptotic time course along with subject-by-subject correlation in the extent of movement and LLR adaptation.

Taken together, the results indicate that the arm’s LLR expresses a wide range of capabilities that reflect knowledge of limb dynamics. Note there are a number of other capabilities reflecting knowledge of limb dynamics which were not discussed at length such as coordinating actions across different effectors (Cole et al., 1984; Dimitriou et al., 2012; Omrani et al., 2013) and predictive responses (Hore and Vilis, 1984; Crevecoeur and Scott, 2013). Unfortunately, the neural basis for these other capabilities is effectively unexplored whereas most of the preceding material has an analogous physiological study. The following sections will describe how primary motor cortex, reticular formation, and cerebellum may contribute to LLR’s knowledge of limb dynamics.

## Contribution by the primary motor cortex

Primary motor cortex (M1) is the medio-lateral strip of cerebral cortex immediately rostral to the central sulcus and marks the beginning of frontal cortex (Porter and Lemon, 1993). It is well-known that M1 provides an important contribution to voluntary control. Changes in its activity precedes movement onset, co-evolves with arm muscle activity (Cheney and Fetz, 1980; Morrow and Miller, 2003), and broadly mirrors the arm’s biomechanics (Scott and Kalaska, 1997; Scott et al., 2001; Cherian et al., 2013). The tight association of M1 activity and voluntary movement is enabled by M1’s substantial descending projection (via the corticospinal track) onto spinal networks that engage the limb’s musculature (Cheney and Fetz, 1980; Park et al., 2004). A wealth of information also indicates that M1 contributes to the LLR. M1 receives rich innervation by somatosensory inputs (via the dorsal column pathway) (Asanuma, 1975) and shows fast activation to perturbations of the wrist, elbow, or shoulder (Conrad et al., 1975; Evarts and Tanji, 1976; Suminski et al., 2007; Herter et al., 2009). Furthermore, an unambiguous linkage to LLRs is established for those M1 neurons with a rapid response to limb perturbations and that direct synapse onto spinal motor neurons (Cheney and Fetz, 1984).

**Figure 5:**
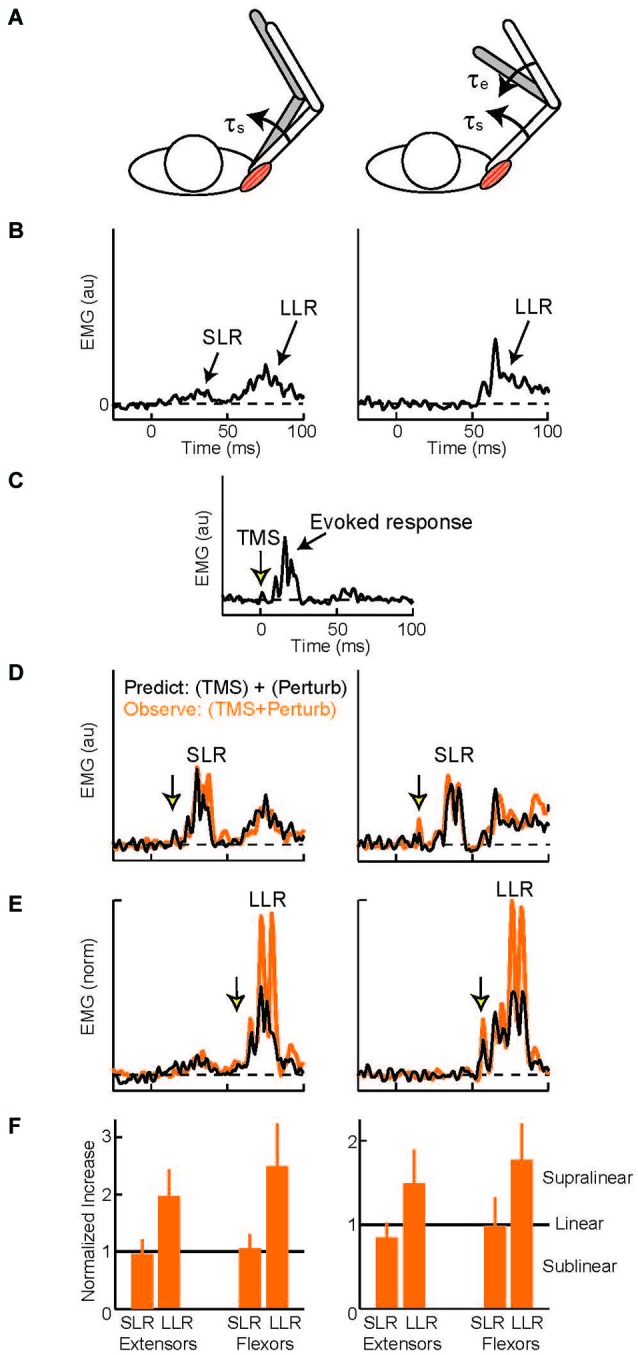
**Evoked muscle activity to perturbation torques and transcranial magnetic stimulation. (A)** Torque perturbations applied to the arm, a shoulder flexion torque which displaced the shoulder joint (left cartoon) and shoulder + elbow flexion torque which only displaced the elbow (right cartoon). **(B)** Evoked activity of the shoulder extensor muscle during shoulder displacement (left panel) and pure elbow displacement (right panel), 0 ms is perturbation onset. Data from a representative subject **(C)** Evoked activity in the shoulder extensor to a single TMS pulse, 0 ms is TMS onset. **(D)** TMS pulse timed to occur during the SLR with shoulder displacement (left panel) and pure elbow displacement (right panel). Orange trace is the observed muscle activity to the combined stimulus; black trace is the predicted response, summed activity to the separate perturbation and TMS stimuli. **(E)** TMS pulse timed to occur during the LLR. Same format as above. **(F)** Group data for the two shoulder muscles. Data normalized to predicted response so values equal to 1 equal linearity whereas values above 1 indicate superlinearity and evidence of a common cortical circuit. Figure modified with permission from Pruszynski et al. (2011a).

Several experimental methods acutely elevate or attenuate the activity of M1 and produce similar effects on the arm’s LLRs. Relatively tonic changes in excitability (lasting several minutes) can be induced by repetitive transcranial magnetic stimulation (TMS; Tsuji and Rothwell, 2002). Single pulses of TMS can also be timed to impact LLRs during a particular perturbation trial. The most common paradigm (Day et al., 1991) compares the magnitude of three muscle responses: evoked by muscle stretch (Figures 5A,B), evoked by a TMS pulse (Figure 5C), and evoked by the two stimuli applied together(Figures 5D,E). If the neural circuits which generate the response to muscle stretch are separate from those which generate the response to a TMS pulse then applying the two stimuli together should yield a response equal to the linear sum from the two separate stimuli: (A) + (B) = (A+B). Alternatively, if the two stimuli are processed through a shared cortical circuit then interactions within that circuit should create a net response different from the sum of the separate responses: (A) + (B) ≠ (A+B). Independence of the generative circuits is evident in the shoulder’s SLR to shoulder muscle stretch (Figures 5B,D, left panels)—no difference from the linear sum—which makes intuitive sense as SLR reflects spinal circuitry and the TMS evoked response depends on cortical circuitry. Evidence of a shared cortical circuit is obtained when the TMS pulse is timed to occur within the shoulder muscle’s LLR to shoulder muscle stretch (Figures 5B,E, left panels) (Pruszynski et al., 2011a). Here the response is substantially larger than the linear sum. Similar observations have been made for finger, wrist, and elbow muscles indicating that M1 generally contributes the LLRs of the upper limb (Day et al., 1991; Lewis et al., 2004, 2006). TMS likely potentiates the LLR by activating cortical circuitry rather than a subcortical target of M1. This inference is justified by the fact that electrical stimulation applied over the scalp (transcranial electric stimulation, (TES)) preferentially excites the descending cortical axons (not the cortical circuitry activated by TMS) and fails to potentiate LLRs (Day et al., 1991).

Despite extensive research showing that primary motor cortex supports LLRs and that LLRs have knowledge of the arm’s biomechanics, there are relatively few studies on M1 contributing this ability to LLRs. Here we discuss the available evidence. One recent study examined multi-joint LLRs of shoulder muscles using the TMS paradigm described above (Pruszynski et al., 2011a). LLRs in shoulder muscles are evoked when displacing only the elbow joint which is appropriate to counter the underlying shoulder-elbow torque that caused elbow motion (see earlier section). These responses must be driven by sensory information from muscles crossing the elbow since the shoulder muscles are not stretched by elbow motion. Accordingly, by timing the TMS pulse to coincide with the shoulder’s LLR during elbow displacement one can test whether the “elbow afferent-to-shoulder muscle” circuit involves M1. For both shoulder extensor and flexor muscles the observed response to elbow displacement and TMS was greater than the linear sum to the two separate stimuli (Figures 5E,F right panels) whereas activity during the SLR was equal to the linear sum (Figures 5D,F right panels). Hence, M1 contributes to multi-joint integration in the LLR appropriate for the arm’s dynamics.

A complimentary experiment was conducted on awake behaving monkeys (Pruszynski et al., 2011a). Recordings of individual M1 neurons were obtained as the animal countered torque pulses applied to its elbow and shoulder. From the entire set of neurons responding to the torque perturbations, a subset was selected which had “shoulder-muscle”-like activity during postural maintenance. These “shoulder muscle”-like neurons were analyzed with the torque comparisons previously described for shoulder muscles. Applied shoulder torque and elbow torques caused a similar initial displacement of the shoulder joint and stretch of the shoulder muscle. If the M1 neurons were driven exclusively by shoulder muscle afferents then they would express a similar burst of activity to the two perturbations. Alternatively, if M1 supports the differential activity observed in the shoulder’s LLR then the neurons should respond more vigorously to the shoulder torque than elbow torque perturbation. Differential M1 activity was observed. A second set of comparisons applied shoulder + elbow torque to cause an initial displacement of just the elbow joint. If the M1 neurons were driven exclusively by shoulder sensory information then they should not respond differently to different direction of elbow motion. Instead, the resulting bursts of M1 activity was greater for elbow motions that required increased shoulder muscle activity to counter the underlying torque, the same pattern observed in the LLRs of shoulder muscles. So in both sets of comparisons, the M1 neurons expressed patterns of activity consistent with a representation of the arm’s mechanical properties. Moreover, M1 neurons expressed this activity pattern 8–20 ms earlier than the same pattern expressed in shoulder muscles consistent with the known conduction delay from motor cortex to the motor periphery.

TMS has been utilized in a different paradigm to test whether M1 contributes to LLR’s knowledge of limb dynamics (Kimura et al., 2006). Here researchers use a strong TMS pulse to induce a prolonged “silent period” following the initial burst of muscle activity. The late phase of the silent period is dominated by cortical inactivation (Ziemann et al., 1993; Brasil-Neto et al., 1995) which blunts M1’s sensory-to-motor processing, and, consequently, immediate contribution to the LLR. This paradigm was first utilized to study reflex modulation while subjects reached to a target placed within a lateral force field (discussed in the previous section, Kimura et al., 2006). LLRs were occasionally elicited prior to the hand entering the force field and these responses were upscaled to compensate the impending lateral force: flexor LLRs were upscaled prior to an expected lateral force requiring flexor compensation, extensor LLRs were upscaled prior to a expected lateral force requiring extensor compensation. On a random number of trials, the researchers applied the strong TMS pulse just prior to the arm displacement. The perturbation still elicited a LLR from the arm muscles, but they were no longer scaled to the upcoming lateral force. Note that the interference was not a general feature of motor neuron quiescence since scaling of LLRs was not abolished during a silent period induced by electrical stimulation of the brachial plexus. To reiterate, temporary blockage of primary motor cortex abolished the scaling of the LLR but not its presence. From this result, the authors posit that primary motor cortex does not generate the LLR but alters its sensitivity. Although this hypothesis runs somewhat counter to a wealth of information, the basic finding has been replicated. Healthy subjects exhibit upscaled LLRs when maintaining their limb posture in a normal environment compared to postural maintenance in a very stiff environment where their motor effects are clamped. LLR scaling to these two environments is abolished during a TMS-induced silent period (Shemmell et al., 2009). Stability-related modulation of LLRs is also absent following cortical stroke (Trumbower et al., 2010), though these individuals express very small responses, unlike the original study, which complicates a direct comparison.

The few studies described in this section provide strong positive evidence that primary motor cortex contributes to the biomechanical knowledge expressed by the arm’s LLRs.

## Contribution by the reticular formation

Reticular formation (RF) is a collection of nuclei spanning the brainstem and which contribute to a wide variety of functions including sensori-motor control (Kuypers, 1981 and Baker, 2011 for review). RF plays a significant role in upper limb behavior as indicated by its prominent descending tract (Lawrence and Kuypers, 1968), innervation of spinal motor neurons controlling muscles throughout the upper limb (Davidson et al., 2007; Riddle et al., 2009), and active modulation during movement and planning stages of self-initiated reaching (Buford and Davidson, 2004; Schepens and Drew, 2004). Studies of individual RF neurons during postural perturbations are extremely limited. The one study on behaving cats demonstrated rapid bursts of RF activity to a foot drop (Stapley and Drew, 2009). Although this activity had an unclear relation to biomechanics or muscle activity, RF likely enables basic control since decerebrate cats have semi-stable stance and normal force reactions to platform displacement (Honeycutt and Nichols, 2010).

**Figure 6:**
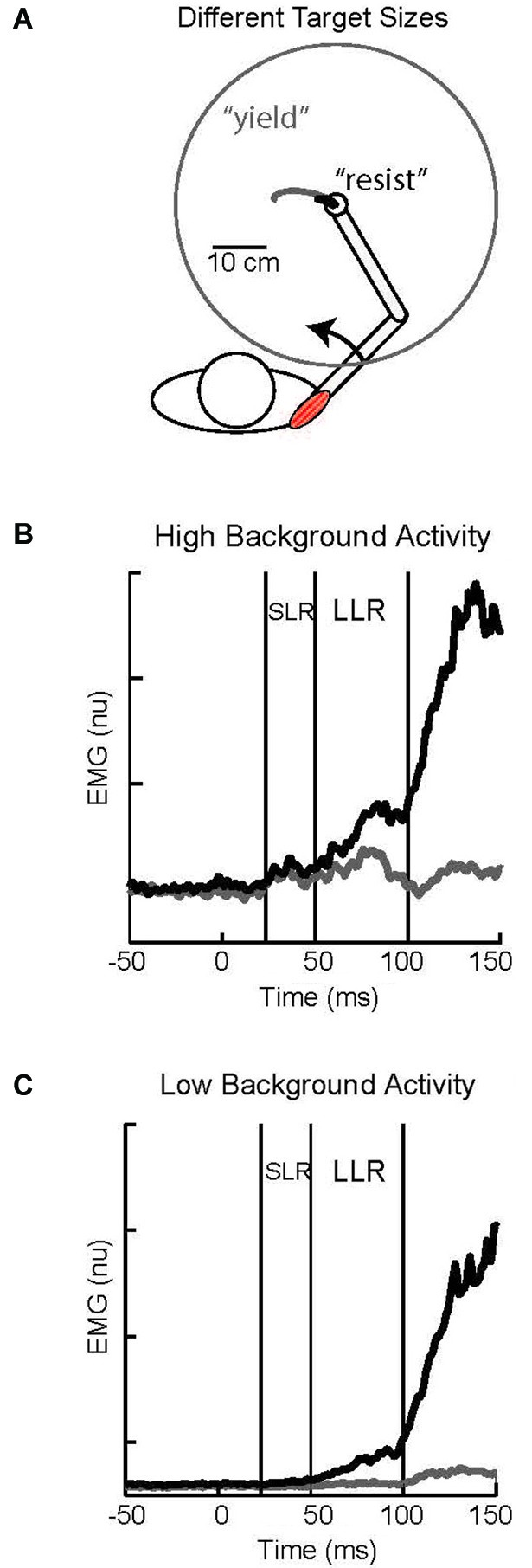
**Task-dependent change in LLR. (A)** Cartoon of subject responding to an imposed shoulder torque. Maintaining the hand within a small target requires a vigorous response and is analogous to a “resist” instruction; the black trace depicts the small displacement of the hand to the perturbation. Maintaining the hand within a large target requires a weak response and is analogous to a “yield” instruction; the gray trace depicts the large displacement of the hand to the perturbation. **(B)** Evoked shoulder activity while the muscle had a high level of background activity from countering a constant opposing load; black and gray traces correspond to the small and large target conditions, respectively.** (C)** Evoked shoulder activity while the muscle had a low level of background activity as its antagonist countered a constant opposing load. Figure modified with permission from Kurtzer et al. (2014).

Larger LLRs occur when subjects attempt to “resist” a perturbation than “yield” to the perturbation. Identified in the earliest studies of the LLR (Hammond, 1956) this capability has spawned considerable research as a clear example of reflex modulation to voluntary goals (Crago et al., 1976; Colebatch et al., 1979; Jaeger et al., 1982; Lee and Tatton, 1982; Calancie and Bawa, 1985; Capaday et al., 1994; Lewis et al., 2006; Pruszynski et al., 2008; Nashed et al., 2012). A powerful paradigm for studying this phenomenon involves visual targets whose location and size, rather than verbal instructions, communicates how subjects should respond (Figure 6A). A small target centered on the hand would require a vigorous response (analogous to “resist”) to adequately counter a perturbation and return to the target area. Conversely, a large target centered on the hand would only require a weak response (analogous to “yield”) to adequately counter a perturbation and remain within the target area. Accumulating evidence indicates that the task-dependent change in LLR activity is due to the temporal overlap of two different responses, a task-dependent response and an automatic response (Rothwell et al., 1980; Lewis et al., 2006; Pruszynski et al., 2011b), rather than the scaling of single process. This is evident in the described paradigm by pairing a small or large visual target with a background load that requires either compensation by the stretched muscles or compensation by its antagonist (and minimum activation by the stretched muscle). With high background muscle activity and a large target the muscle displays bursts of activity within the SLR and LLR epochs whereas there is almost no evoked activity with low background activity and the large target (Figures 6B,C); such changes in response magnitude with background activity is termed “automatic gain scaling” (Matthews, 1986; Pruszynski et al., 2009). In contrast, a nearly constant increase in LLR activity is observed with the small target relative to the large target regardless of background muscle activity (Figures 6B,C). This pattern is consistent with the addition of a task-dependent component to an automatic component (Pruszynski et al., 2011b).

RF is a candidate generator for the task-dependent response. A direct link between RF and LLR’s task-dependency is stymied by a complete lack of neural recordings during this behavior, but an indirect link can be made via the “StartReact”. StartReact is the ultra-fast initiation of a planned action by a startling stimulus (typically a loud tone, 120 dB) (Valls-Solé et al., 1999; for review see Carlsen et al., 2011). Arm muscle activity during StartReact occurs ≈70 ms after the startling stimulus compared to >100 ms for a non-startling stimulus. This faster than normal reaction likely reflects the engagement of RF since these circuits underlie the protective startle response (Yeomans et al., 2002) and subjects suffering a cortical stroke (Honeycutt and Perreault, 2012) or degenerated corticospinal tracts (Nonnekes et al., 2014) will have delayed voluntary reactions but normal onset of StartReact.

The relation between LLR’s task-dependency and StartReact was recently tested (Ravichandran et al., 2013). Subjects were instructed to quickly initiate an elbow movement following an auditory cue. On a random set of trials, their limb was perturbed or a loud sound was presented. The two stimuli caused a similar pattern of activity in the LLR epoch and evoked similar activity of the neck muscle sternocleidomastoid (an indicator of startle). Hence, there is an impressive similarity between the task-dependent response and StartReact.

Before proceeding, it is important to ask whether task-dependency of LLRs could reflect a neural substrate other than RF. Primary motor cortex is a likely candidate for reasons already elaborated. Its perturbation evoked activity also expresses task-dependent changes that parallel the set-dependent changes in upper limb LLRs (Evarts and Tanji, 1976). Recent studies have confirmed that task-dependency is commonly expressed across the population of M1 neurons, though in a more complex manner than earlier supposed, and has the appropriate timing to contribute to the observed muscle responses (Omrani et al., 2014; Pruszynski et al., 2014). However, transient suppression of M1 by a powerful TMS pulse does not diminish the LLR’s magnitude during the “resist” instruction (Shemmell et al., 2009). This suggests that the supraspinal generator of LLR’s task-dependent component is downstream from M1. RF is the most likely candidate given its general role in posture control and its specific role in StartReact.

**Figure 7:**
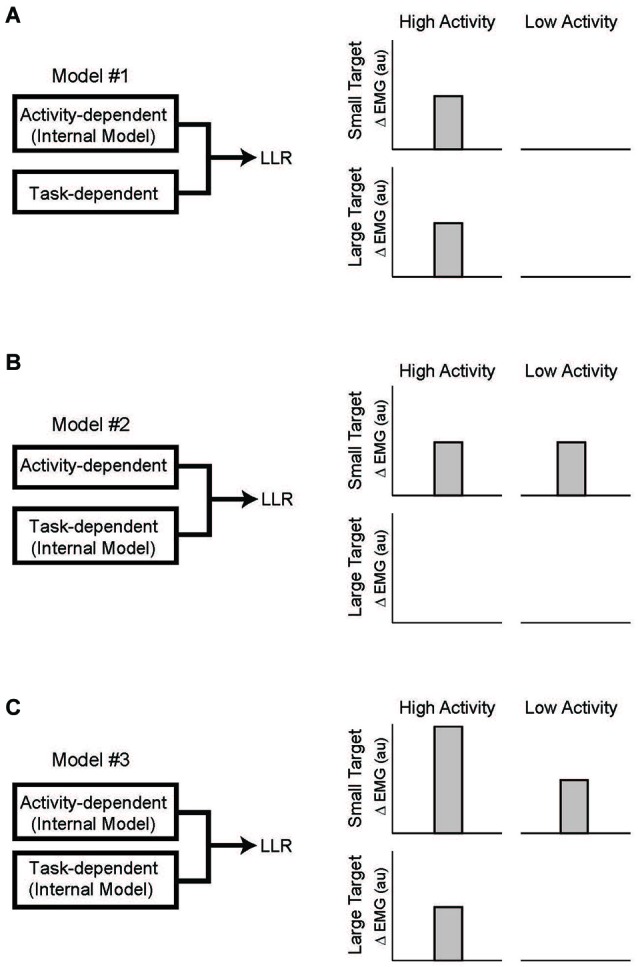
**Testing which component of the LLR utilizes knowledge of limb dynamics. (A)** Left panel depicts a simple model of LLR comprised of two functional component: an automatic component scaled by background muscle activity and a task-dependent component scaled by target size. Right panel depicts expected pattern of LLR if only the automatic component utilized an internal model of limb dynamics. Expression of that information (i.e., a differential response to the pair of perturbations) would be evident during high background activity of the muscle but would not change with target size. **(B)** If only the task-dependent component utilized an internal model of limb dynamics than expression of that information would be evident with a small target requiring a vigorous response and not change with background activity of the muscle.** (C)** If both the automatic and task-dependent components utilized an internal model of limb dynamics than expression of that information would co-vary with background activity and target size. Figure modified with permission from Kurtzer et al. (2014).

**Figure 8:**
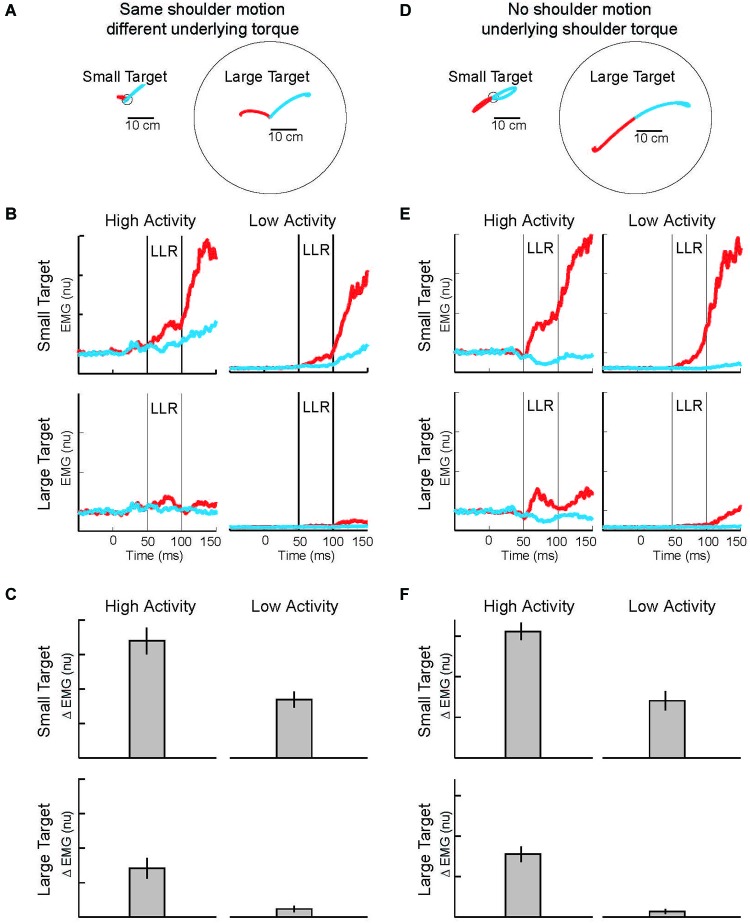
**Modulation of LLR to target size and background muscle activity. (A)** Torque perturbations applied to the arm, a shoulder flexor torque (red arm) and an elbow extensor torque (blue arm). Red and blue traces show exemplar hand paths resulting from the two torque perturbations during presentation of a small target or large target. **(B)** Group average of shoulder extensor muscle activity evoked by the shoulder flexor torque (red) and elbow extensor torque (bue). The four panels display data during the four combinations of background muscle activity and target size. **(C)** Differential activity of the LLR to the pair of perturbations (shoulder flexor torque and elbow extensor torque) given the four combinations of target size+background muscle activity (compare to predictions in Figure 7). **(D)** Torque perturbations applied to the arm, a shoulder + elbow flexor torque (red arm) and a shoulder + elbow extensor torque (blue arm). Red and blue traces show hand paths resulting from the perturbations, same format as above. **(E)** Group average muscle activity evoked by the shoulder + elbow flexor torque (red) and shoulder + elbow extensor torque (bue). **(F)** Differential activity of the LLR to the pair of perturbations (shoulder flexor+elbow flexor torque and shoulder extensor+elbow extensor torque) given the four combinations of target size+background muscle activity (compare to predictions in Figure 7). Figure modified with permission from Kurtzer et al. (2014).

With the link between task-dependency of the LLR and RF tentatively established, we now ask whether the task-dependent component, the automatic component, or both possesses knowledge of the arm’s dynamics (Figure 7). The one study which has examined this important question (Kurtzer et al., 2014) used the 2 × 2 experimental design described above. Subjects were presented with either a small (radius = 2 cm) or large (radius = 30 cm) requiring a vigorous or weak corrective response along with background loads requiring low or high levels of pre-perturbation activity of the examined muscle, a shoulder extensor. The only experimental difference is that two pairs of torque perturbations were utilized: (1) shoulder flexion torque and elbow extension torque to induce similar amounts of initial shoulder flexion (Figure 8A); (2) shoulder − elbow flexion torque and shoulder − elbow flexion torque to induce flexion or extension of just the elbow (Figure 8B). Differential activity to each pair of perturbations is positive evidence for the LLR’s knowledge of limb dynamics (see Figure 2). If knowledge of limb dynamics was only expressed by the LLR’s automatic component then differential activity should be present with high background activity and absent without background activity, i.e. differential activity would only change with the muscle’s background activity (Figure 7A). If knowledge of limb dynamics was only expressed by the LLR’s task-dependent component then the magnitude of differential activity should be present with the small target and absent with the large target, i.e. differential activity would only change with the target size (Figure 7B). Lastly, if knowledge of limb dynamics was expressed by both the automatic and task-dependent component of the LLR then the magnitude of differential activity should increase with high background activity and the small target (Figure 7C).

Evoked muscle activity to shoulder displacement (the first pair of perturbations) was highly modulated by target size and background muscle activity (Figures 8B,E). Similar to previous studies, the greatest LLRs occurred with the small target and high background combination (top-left panel) which presumably recruits both components whereas very weak LLRs occurred with the large target and low background combination (bottom-right panel) which presumably recruits neither component. Response magnitudes between these extremes occurred for the large target/high background combination and the small target/low background combination presumably because the automatic or task-dependent component is selectively recruited, respectively. The critical issue is how the differential activity to the shoulder torque and elbow torque perturbations changes with target size and background activity, the difference between the red and blue traces in each panel. The differential activity clearly changes with target size and background activity and has the greatest magnitude when both components presumably contribute, compare Figures 7C, 8C.

Complementary results were obtained with the pair of torque perturbations causing motion of just the elbow (Figure 8D). Again, the greatest LLRs occurred with the small target and high background combination (top-left panel), the smallest LLRs occurred with the large target and low background combination (bottom-right panel), and response magnitudes between these extremes occurred for the large target/high background combination and the small target/low background combination. As before, the critical issue is how differential activity to the two perturbations (leading to excitatory and inhibitory effects) changes with target size and background activity, the difference between the red and blue traces in each panel. The differential activity is greater during conditions with a small target and conditions with high background activity. The greatest differential magnitude occurs during the small target/high background combination when both the automatic and task-dependent components would contribute, compare Figures 7C, 8F.

Taken together, the results indicate that the task-dependent component and automatic component of the LLR utilize knowledge of limb dynamics. Given the indirect link between task-dependency in the LLR and RF we tentatively conclude that RF contributes to this internal model. A direct test is lacking but would involve neural recordings from this structure. Another possibility is TES as this activates the descending axons of M1 (not its laminar circuitry) and would engage RF though a serial connection or a TMS silence period paradigm that suppresses cortical processing. Clearly, a great deal of work is needed before a definitive conclusion can be made.

## Contribution by the cerebellum

The cerebellum is a massive and highly elaborated subcortical structure which provides a distinct contribution to sensori-motor control (Manto, 2002). In the broadest strokes, the cerebellum is not necessary for either sensation or action, but is critical to motor coordination. It receives somatosensory information from the motor periphery as well as information from motor-related cerebral, brainstem, and spinal networks. Damage to the cerebellum can result in a variety of abnormalities in self-initiated arm movements including improper timing, scaling, and launch direction along with pronounced tremor as the hand nears its target. These problems are present for actions performed at a single joint, like the elbow or wrist (Hallett et al., 1975; Brown et al., 1990; Manto et al., 1994), but are relatively mild compared to the disturbed behavior of the unconstrained arm (Holmes, 1939; Goodkin et al., 1993; Bastian et al., 1996) where limb motion becomes irregular and inconsistent. Cerebellar damage also impairs the ability to actively stabilize the shoulder when attempting fast elbow-only movements (Boose et al., 1999) indicating that subjects inadequately anticipate the arm’s multi-joint dynamics, rather than an issue specific to producing multi-joint movement trajectories. Over-compensation and under-compensation also show that inaccuracy is not due to an inability in producing adequate phasic force to counter intersegmental dynamics (Bastian et al., 2000).

The prominent connection between the cerebellum and primary motor cortex (Middleton and Strick, 1998) and the prominent role of primary motor cortex for the LLR (Pruszynski and Scott, 2012) suggests that the cerebellum has an important role for LLRs. (Note that the cerebellum also provides input to the RF and almost certainly modulates its action (Asanuma et al., 1983)). Neurons in the dentate and interposed output nuclei of cerebellum respond to limb perturbations over a range of times (Strick, 1983), the earliest bursts of cerebellar activity could impact M1 activity within the long-latency epoch. Cerebellum does influence M1’s processing of somatosensory information since a cooling probe applied to cerebellar output nuclei depresses reflex-related activity of M1 (Meyer-Lohmann et al., 1975). Moreover, cerebellar cooling results in the limb behaving like an underdamped spring with sustained oscillations over the goal target (Vilis and Hore, 1980). Evidence is mixed whether the cerebellum alters the LLR of stretched upper limb muscles. Cerebellar cooling did not alter the LLR in the stretched elbow muscle. Rather the shortened elbow muscle had a delayed and sustained antagonist burst which initiated the oscillatory movement. Researchers have also reported lowered (Marsden et al., 1977) and heightened long-latency responses (Friedemann et al., 1987) in the hand muscles. Note that these studies examined the LLRs when controlling motion at a single joint, a situation known to be less compromised than multi-joint control. To date, only one study has examined if cerebellar damage alters the arm’s LLRs during multi-joint control (Kurtzer et al., 2013) and compromised the LLR’s knowledge of limb dynamics.

**Figure 9:**
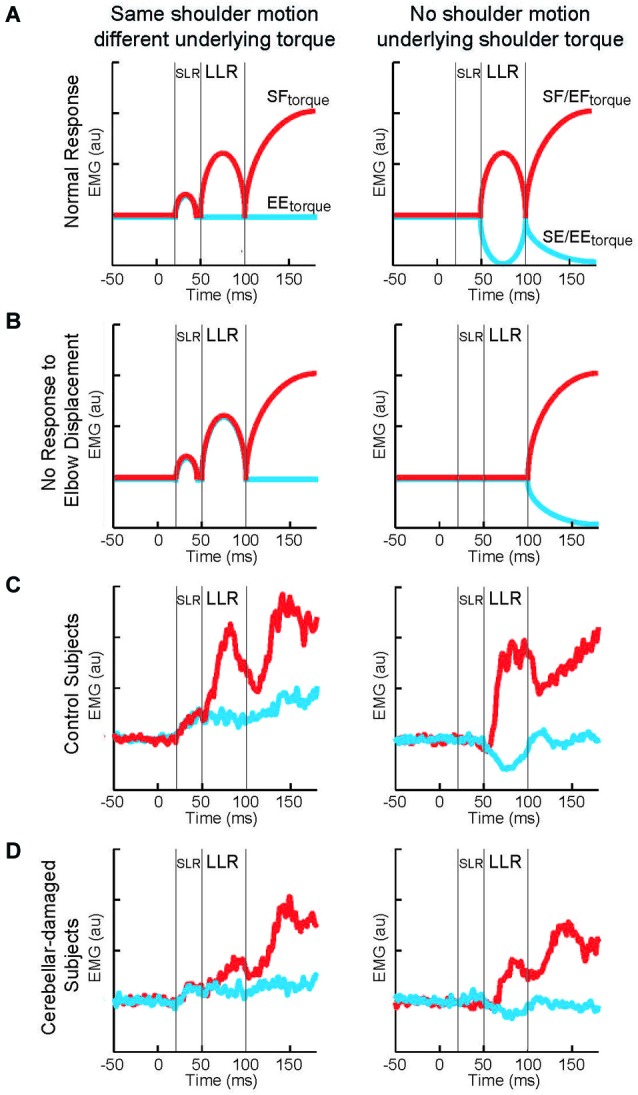
**Long-latency reflexes during cerebellar damage. (A)** Cartoon of evoked activity from the shoulder extensor by healthy subjects. Panels on the left indicate responses to shoulder displacement caused by a shoulder flexor torque (red) and an elbow extensor torque (blue). Panels on the right indicate responses to elbow displacement caused by a shoulder + elbow flexor torque (red) and an shoulder + elbow extensor torque (blue). **(B)** Cartoon of predicted shoulder muscle activity if cerebellar damage eliminates the ability to integrate multi-joint muscle. **(C)** Group average of shoulder extensor muscle activity by a group of healthy subject. **(D)** Group average of shoulder muscle activity by a group of subjects suffering cerebellar damage. Figure modified with permission from Kurtzer et al. (2013).

To test if cerebellar damage compromises the knowledge of limb dynamics utilized by the arm’s LLR, the authors employ a paradigm described in the previous sections (Figures 2, 3). Subjects maintained their arm in a steady posture while four different torque perturbations were unexpectedly applied. A shoulder flexor torque and elbow extensor torque induced the same amount of initial shoulder flexion whereas a shoulder-elbow flexion torque and shoulder-elbow extension torque induced pure elbow flexion and pure elbow extension, respectively. These torque combinations tested whether the shoulder extensor’s LLR was driven by shoulder motion only or by motion of both joints appropriate to counter the underlying torque. The participants in the experiment included individuals who suffered cerebellar damage leading to ataxic arm behavior and healthy matched controls. If the pattern and magnitude of LLRs were entirely independent of the cerebellum, then cerebellar damaged individuals would express normal LLRs (Figure 9A). Alternatively, if knowledge of limb dynamics depends entirely on the cerebellum, then their shoulder LLRs would continue to respond to the local shoulder motion but fail to respond to elbow motion (Figure 9B); that is, the LLRs would show the same simple pattern of response exhibited by the SLR.

The motor behavior of cerebellar-damaged individuals exhibited the characteristic oscillatory and inaccurate arm motion to limb perturbations. Their LLRs were also altered from normal but in an unexpected way. Both healthy and clinical subjects had greater LLRs in the stretched shoulder extensor when shoulder displacement was induced by shoulder flexor torque vs. elbow extensor torque (Figures 9C,D, left panels). Healthy and clinical subjects also had excitatory shoulder LLRs when their elbow was displaced into flexion and inhibitory LLRs when their elbow was displaced into extension (Figures 9C,D, right panels). Accordingly, cerebellar-damaged individuals expressed a pattern of multi-joint integration appropriate for the arm’s intersegmental dynamics.

The main difference from normal was much smaller LLRs. The smaller LLRs in the cerebellar-damaged group did not reflect a general change in reflex excitability as their SLR was not decreased from normal. Nor did smaller LLRs reflect a less vigorous motor set since their voluntary response (>100 ms) was not decreased from normal. Nor was there greater downscaling of LLRs to a sub-set of perturbations. Rather the cerebellar group had the same relative magnitudes of LLRs to the perturbations as the normal group.

The conserved pattern of LLR activity indicates that the knowledge of limb dynamics used to generate LLRs is housed outside of the cerebellum. The smaller level of activity suggests that its overall sensitivity to limb displacement is modulated by the cerebellum. A similar conclusion has been made by a several researchers (Holmes, 1939; MacKay and Murphy, 1979; Jo and Massaquoi, 2004).

One can make a reasonable *post hoc* explanation for the lowered LLRs with cerebellar damage. Cerebellum enables a broad number of motor abilities that rely on predicting future states of the body based on current sensory information, ongoing motor commands, and a representation of limb mechanics (for review see Bastian, 2006), i.e. a forward model. Recordings of single Purkinje neurons while the monkey moved its arm against different loads demonstrate that cerebellar activity is linked to the predicted state of the arm not the exerted motor commands (Pasalar et al., 2006). Such forward models allow fast feedback control of a system with time-delays, like our body and nervous system. If the predicted sensory states are noisy and inaccurate, like with cerebellar damage, then feedback gains must be decreased in order to ensure stability. This reasoning has accounted for the altered behavior of lift-grip actions made in a low gravity environment (Crevecoeur et al., 2010). A recent study also found that single-joint arm movements by cerebellar damaged subjects were consistent with lowered feedback gain (Bhanpuri et al., 2014). In addition, cerebellar damage has been shown to degrade the predictive ability of fast feedback control including scaling the initial leg muscle response to the amplitude of surface displacement (Horak and Diener, 1994) and cerebellar cooling eliminates the ability to generate early antagonist responses to a pulse perturbation (Hore and Vilis, 1984). Taken together, the cerebellum may use prediction accuracy of its forward models to gain modulate the neural pathways providing knowledge of limb dynamics to the LLR.

## Possible contribution by other neural substrates

The three previous sections discussed how primary motor cortex, RF, and cerebellum enable the LLR’s compensation of arm’s biomechanics. The focus on these three brain regions was not intended to exclude other possible contributors, but describe the relatively few physiological studies that are directly relevant. Neural pathways which could contribute to this capability but have not been tested include the basal ganglia, red nucleus, additional cortical areas, and spinal cord. Basal ganglia is a likely candidate as it is strongly linked to primary motor cortex and RF (Middleton and Strick, 2000). Moreover, disorders of basal ganglia are linked to alterations in LLRs such as an increased response magnitude paralleling the well-known increase in limb rigidity (Tatton and Lee, 1975; Rothwell et al., 1983) and an inability to alter LLRs to the perturbation context such as platform tilt when standing with or without a hand-hold (Schieppati and Nardone, 1991, also Horak et al., 1992). Red nucleus likely plays an important role in the LLRs of non-human primates via its substantial descending tract to the spinal cord (Lawrence and Kuypers, 1968) and generally similar activity patterns as M1 neurons (Cheney et al., 1991). However, the rubrospinal tract is quite small in humans (Nathan and Smith, 1982) and is not expected to provide significant direct contributions to motor output including the LLR. Several motor cortical regions, in addition to primary motor cortex, could be involved in the LLR by either projecting to M1, brainstem or spinal targets. This includes supplemental motor area which appears to modulate the automatic component of LLR via its connection with M1 and may engage the RF and its task-dependent component of LLR (Hummelsheim et al., 1986; Dick et al., 1987; Spieser et al., 2013). Lastly, group II-spinal circuits most likely contribute to the LLR (Hendrie and Lee, 1978; Lourenço et al., 2006; Meskers et al., 2010) and electrical stimulation of the peripheral nerves has revealed that group II afferents make multi-muscle connections (Lourenço et al., 2006). Future studies should uncover the efficacy and pattern of these connections during more naturalistic limb displacements. In sum, there are variety of neural pathway which could provide complimentary or distinct functions to the LLR that enable it to account for the arm’s biomechanics.

## Conclusion

A significant body of work has explored the capabilities of the LLR. Knowledge of limb dynamics is a core capability of LLRs and allow a degree of motor sophistication that rivals planned voluntary actions (Scott, 2004; Pruszynski and Scott, 2012). The material reviewed here considers three supraspinal circuits which may support this function: primary motor cortex, RF, and cerebellum.

The most direct and convincing evidence is that primary motor contributes to the LLR’s knowledge of limb dynamics. This is consistent with M1’s strong link to the motor periphery, LLRs, and motor adaptation. Although there are relatively few studies on this topic, they utilize neural recordings and non-invasive brain stimulation, the data is unambiguous and the logic is straight-forward. Taken together, it can be concluded that primary motor cortex provides knowledge of limb dynamics used by the LLR.

The RF is another natural candidate given its sensory and motor pathways. Although there is no direct evidence (given the complete lack of neural recordings) we can make reasonable inferences on the neural basis of StartReact and its association with task-dependency of the LLR. Given this chain of reasoning, the evidence is consistent with a reticular contribution. It can be tentatively concluded that RF provides knowledge of limb dynamics used by the LLR.

The final supraspinal circuit we considered is the cerebellum. This is still another natural candidate given its sensory inflow, efferent connection to primary motor cortex, and critical role in motor coordination. The one study on this topic examined a clinical population. These individuals had the classical signs of ataxia and postural instability yet their LLR had the same pattern of activity as normal. An unaltered motor pattern in a clinical population indicates that the damaged brain area does not directly contribute to that motor pattern. We concluded that the cerebellum scales the gain of neural pathways that provide the structured response of the LLR.

It should be emphasized that the material on this topic is a starting point and not a final chapter. A few outstanding questions in no particular order:

- If multiple supraspinal substrates possess knowledge of limb dynamics, in what ways do they differ?
- Do spinal pathways possess knowledge of limb dynamics?
- What is the neural basis of adapting long-latency reflexes?
- How do the neural circuits represent the different features of the body/environment?
- In what ways does the knowledge of limb dynamics for the long-latency reflex differ from that utilized by self-initiated/voluntary actions?

Answering these questions will greatly enrich our understanding of fast feedback control.

## Conflict of interest statement

The author declares that the research was conducted in the absence of any commercial or financial relationships that could be construed as a potential conflict of interest.
